# Heavy grazing causes plant cluster fragmentation of sparse grasses

**DOI:** 10.1002/ece3.10581

**Published:** 2023-10-05

**Authors:** Zihan Wang, Shijie Lv, Hongmei Liu, Chen Chen, Zhiguo Li, Zhongwu Wang, Guodong Han

**Affiliations:** ^1^ College of Grassland, Resources and Environment Inner Mongolia Agricultural University Hohhot Inner Mongolia China; ^2^ Science College Inner Mongolia Agricultural University Hohhot Inner Mongolia China; ^3^ Forestry Research Institute of Inner Mongolia Autonomous Region Hohhot Inner Mongolia China

**Keywords:** different scales, geo‐statistics, plant cluster fragmentation

## Abstract

*Cleistogenes songorica*, as a clustered grass, is the main grassland flora of the *Stipa breviflora* desert grassland. Some studies have shown that the constructive species of *S. breviflora* (sparse cluster type) is prone to cluster fragmentation; however, research on *C. songorica* is relatively rare. Then will the *C. songorica* plant population (dense cluster type) also have cluster fragmentation under the influence of intense grazing? To answer this question, we used variance analysis and geo‐statistical methods. The spatial distribution of *C. songorica* in *S. breviflora* desert steppe in Inner Mongolia was measured under four grazing intensities (no grazing, CK, 0 sheep·ha^−1^·half year^−1^; light grazing, LG, 0.93 sheep·ha^−1^·half year^−1^; moderate grazing, MG, 1.82 sheep·ha^−1^·half year^−1^; and heavy grazing, HG, 2.71 sheep·ha^−1^·half year^−1^) and four scales (10 cm × 10 cm, 20 cm × 20 cm, 25 cm × 25 cm, 50 cm × 50 cm). We then analyzed *C. songorica* whether fragmentation was present. The results showed that increased grazing intensity is associated with increased density and decreased height, coverage, and standing crop of *C. songorica*. The spatial distribution of *C. songorica* was affected by structural factors, and spatial heterogeneity decreased with increased spatial scale. With increased grazing intensity and spatial scale, the patch area of *C. songorica* gradually increased and tended toward band distribution. In summary, increased grazing intensity and spatial scale led to weakened heterogeneity of *C. songorica* spatial distribution and increased consistency.

## Graphical abstract

Heavy grazing led to the fragmentation of *Cleistogenes songorica*. With increasing grazing intensity and spatial scale, the patch area of *C. songorica* gradually increased and tended toward a band distribution. As grazing intensity and spatial scale increased, the heterogeneity of *C. songorica* spatial distribution weakened and consistency increased.

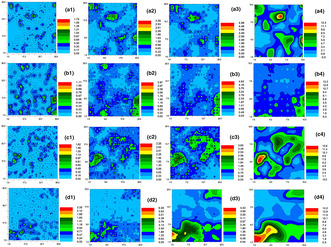

## 1 INTRODUCTION

Grazing is the most direct means of utilizing grassland (Li et al., 2015). The change and succession of grassland vegetation is disturbed by grazing, which impacts the structure and function of the grassland ecosystem more broadly (McNaughton, 1983; Milchunas et al., 1988). Grazing changes the spatial distribution of plants (Dad, 2019). The same plant population will exhibit different spatial patterns under different disturbances (Yang et al., 2011). Spatial heterogeneity is an important indicator for describing the spatial distribution of plant communities (Almeida et al., 2016; Li et al., 2020). Spatial heterogeneity refers to the spatial in‐homogeneity and complexity of a plant community, which is a comprehensive reflection of spatial patchiness and spatial gradient. Spatial heterogeneity is an important feature in evaluating grassland plaque. The grazing behavior of herbivores increases or decreases the spatial heterogeneity of plant populations due to differences in grazing intensity (de Vries & Daleboudt, 1994). For example, sheep grazing caused fragmentation in the original large patches to new smaller patches (Bisigato & Bertiller, 1997). In desert steppe, the spatial heterogeneity of *Stipa breviflora* population is directly proportional to its grazing intensity (Lv et al., 2014), and the scale affects the spatial structure of plant population (Zhang et al., 2021). Spatial distribution strongly depends on differences in spatial scale (Bai et al., 2012). Spatial scale is an important dimension for evaluating changes in plant populations, and the division of different scale ranges is of great significance to the study of spatial distribution (Kleijn & Steinger, 2002).

Desert steppe is a fragile ecological environment (Peng et al., 2013). Minimal precipitation increases the sensitivity of desert grassland to interference by grazing, and its recovery threshold is low (Coupland, 1993). *Stipa breviflora* is a perennial dense grass and constructive species in desert steppe. Studies have found that spatial aggregation can enhance the grazing tolerance of *S. breviflora*, and small isolated clusters are beneficial to the survival of this dominant species under heavy grazing. The spatial distribution of constructive species such as *S. breviflora* has long been of interest to scholars, but the stability of dominant species is also an important research area as it is closely related to ecosystem stability (Lv et al., 2019; Sasaki & Lauenroth, 2011; Wayne Polley et al., 2007).

*Cleistogenes songorica*, as a perennial sparse grass that lives in arid or semiarid ecosystems (Lv et al., 2019). It is the dominant species in desert steppe and has the advantages of being tolerant to cold, drought, and trampling (Chen et al., 2002; Yang et al., 2011). Studies have shown that the stem base diameter of *C. songorica* becomes smaller when disturbed by heavy grazing. It can also adapt to high‐intensity livestock feeding by reducing leaf area and slowing its growth rate to reduce loss from livestock, showing strong grazing avoidance ability (Peng et al., 2013). Therefore, in this paper geo‐statistical analysis was used to study whether increased grazing intensity would cause the fragmentation of *C. songorica* in sparse grasses of the desert steppe in Inner Mongolia, as well as its degree of influence. Three research questions were proposed to solve the problem: (1) How do the basic quantitative characteristics (density, height, coverage, and standing crop) of *C. songorica* change with increased grazing intensity? (2) With increased grazing intensity, does *C. songorica* appear the phenomenon of plant cluster fragmentation? (3) What are the changes in the fragmentation of plant clusters with increased grazing intensity and spatial scale?

## 2 MATERIALS AND METHODS

### 2.1 Study site

The basic quantitative characteristics and standing crop collection of *C. songorica* were carried out in a long‐term grazing experiment in Siziwang Banner (41°46′43.6″ N, 111°53′41.7″ E, elevation 1450 m), Comprehensive Experimental Demonstration Center, Inner Mongolia Academy of Agricultural and Animal Husbandry Sciences. The average annual precipitation is 223 mm and the average temperature is 3.4°C. The soil is sandy loam, and the desert steppe in this region is dominated by *S. breviflora*, *Artemisia frigida*, and *C. songori*ca. The vegetation is sparse, and the composition of plant species is relatively simple. The average height of vegetation is 5 cm.

### 2.2 Experimental design

**FIGURE 1:**
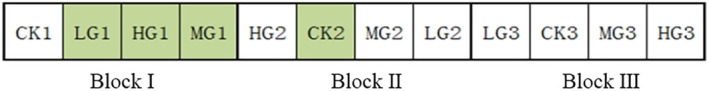
Schematic diagram for the grazing experiment plots. Dark color plots indicate sampling plots. The grazing experiment plots (each ca. 4.4 ha) were arranged in a randomized complete block design, which included four grazing intensity treatments with three repeats at each grazing intensity.

This study used a randomized block design with four divisions of grazing intensity: no grazing CK (0 sheep·ha^−1^·half year^−1^), light grazing LG (0.93 sheep·ha^−1^·half year^−1^), moderate grazing MG (1.82 sheep·ha^−1^·half year^−1^) and heavy grazing HG (2.71 sheep·ha^−1^·half year^−1^). Each treatment plot of grazing intensity was randomly arranged and repeated three times (Figure 1). Since 2004, the annual seasonal grazing period for 2‐year‐old Mongolian sheep is from the beginning of June to the end of November. The management measures in each grazing area were basically the same during the experiment. The daily grazing time was from 6:00 a.m. to 6:00 p.m., and the grazing period was 6 months. Throughout the grazing period, sheep were regularly provided with water and salt.

### 2.3 Vegetation sampling

**FIGURE 2:**
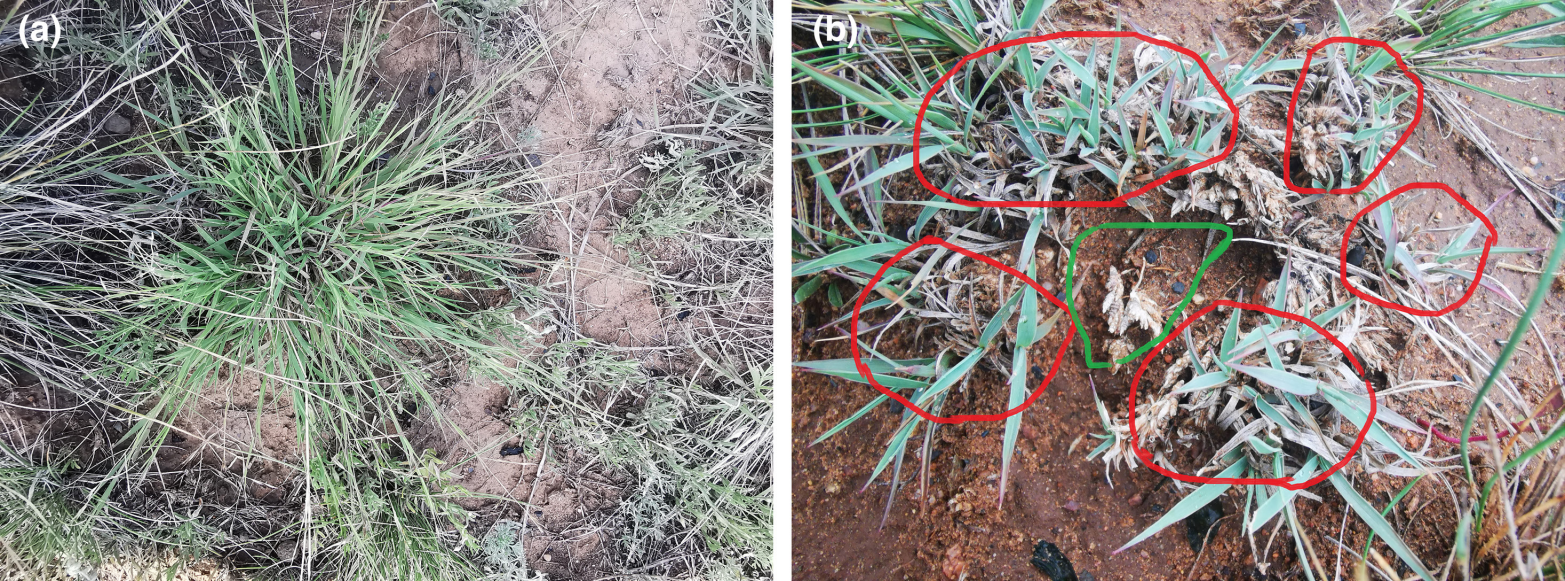
The changes of *Cleistogenes songorica* in CK (a) and HG (b) of desert steppe. The circles indicate clusters of *C. songorica*. The red circle represents the fragmented state of *C. songorica* in the HG plot, while the green circle represents the exposed state of the surface after *C. songorica* in the middle area is eaten.

Early observations in the experiment revealed obvious changes to plant clusters in CK and HG plots (Figure 2). To further investigate the basic quantitative characteristics of the plant population of *C. songorica* in desert steppe, ten 50 cm × 50 cm quadrats were randomly placed in each treatment, and height, coverage, and density of *C. songorica* were recorded in each plot. These were then mowed and placed in a laboratory oven at 65°C to remove water and weigh. The CK plot of block II was selected to avoid the potential edge effect in the CK plot in block I. For different grazing intensities in each sample plot, a representative plot with the same terrain (5 m × 5 m) was selected. The origin coordinate in each sample was defined as the upper left corner of the plot. A 1 m × 1 m sample frame was then placed 25 times in turn, and the spatial position of in the sample recorded using a roll ruler. The *C. songorica* data collection for this study took place on August 15, 2019.

### 2.4 Data analysis

The data for density (cluster/m^2^), height (cm), coverage (%) and standing crop (g/m^2^) of *C. songorica* were subjected to variance homogeneity tests (*p* < .05) under different grazing intensities, and all obeyed normal distribution. The generalized linear model (GLM) was used to test the effect of grazing intensity on the quantitative characteristics of *C. songorica*. The Duncan test (Levene homogeneity test) was used to compare the mean and standard deviation of grazing treatments. The representative sample plot of 5 m × 5 m was divided into 10 cm × 10 cm, 20 cm × 20 cm, 25 cm × 25 cm, and 50 cm × 50 cm, with sample numbers of 2500, 625, 400, and 100, respectively. After transforming the data into square root, it obeys normal distribution, and variance analysis was used to compare changes in density of *C. songorica* under different grazing intensities and spatial scales. In other words, the GLM process was used to compare the density of *C. songorica* under different grazing intensities and spatial scales. SAS 9.4 (SAS Institute Inc.) software was used to perform the variance analysis at *p* < .05 level and SigmaPlot 14.0 (Systat Software, 2011) was used to complete the graphic drawing.

Before geo‐statistical semi‐variogram analysis, the sample data distribution's skewness, kurtosis, and confidence intervals were calculated. If all skewness and kurtosis is contained within the intervals, the sample data is considered to have a normal distribution. Calculations were performed in Excel 2016 (Microsoft Inc.), and the sample data fit within the skewness and kurtosis confidence intervals.

This study used geo‐statistical methods to analyze the spatial heterogeneity of *C. songorica* density in areas of 10 cm × 10 cm, 20 cm × 20 cm, 25 cm × 25 cm, and 50 cm × 50 cm. The optimal fitting model was constructed, then spatial distribution characteristics of the plant population were analyzed using the semi‐variance function method. The Kriging method was used for spatial interpolation, and then the map of *C. songorica* density was drawn according to the semi‐variance function (Matheron, 1963).

The spatial distribution characteristics of *C. songorica* density were selected following the principle of residual variance square and RSS (Residual Sum of Squares), and linear, exponential, spherical, and Gaussian fitting models were selected (Augustine & Frank, 2001). The spatial auto‐correlation ranges were *A*_0_, 3*A*_0_, *A*_0_, and $\sqrt{3}$ *A*_0_, respectively. The parameters of spatial distribution were selected by the semi‐variance function, which mainly includes *C*_0_, *C*_0_ + *C*, and *A*_0_. The nugget value *C*_0_ is also called nugget variance and indicates spatial variation caused by random factors. The base value *C*_0_ *+ C* reflects the spatial variation caused by structural and random variation factors. Structural variance *C*/(*C*_0_ *+ C*) is the spatial change caused by structural factors (such as soil, topography, geomorphology, etc.). *C*/(*C*_0_ *+ C*) is the proportion of spatial variability of structural factors in the total variation. A value of *C*_0_/(*C*_0_ *+ C*) < 25% indicates that spatial heterogeneity is mainly structural variation. 25% ≤ *C*_0_/(*C*_0_ *+ C*) < 75% indicates that the variable has medium spatial correlation. *C*_0_/(*C*_0_ *+ C*) ≥ 75% indicates weak spatial correlation and that random variation is dominant. The variable *A*_0_ is used to indicate that the range of spatial correlation of regionalized variables is within the variable. The variables are correlated and the space is not independent. In addition to the range, the variables are irrelevant and independent in space. Statistical analysis was performed using GS+ software (Version 9, Gamma Design software, 2014).

## 3 RESULTS

### 3.1 The quantitative characteristics of *Cleistogenes songorica*

**FIGURE 3:**
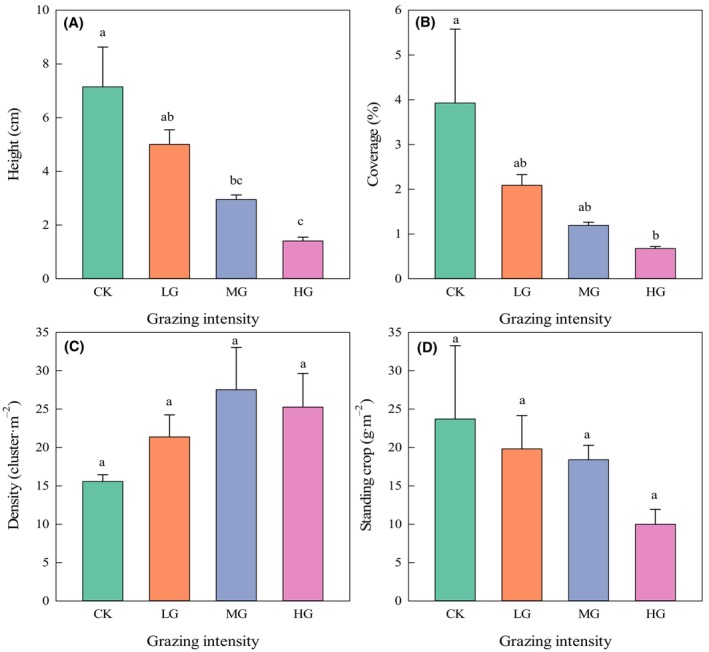
Effects of grazing on the basic quantitative characteristics of *Cleistogenes songorica* in the grazing treatment plots. Mean values (±SE; *n* = 30) of (A) height, (B) cover, (C) density, and (D) standing crop. The results were variance analysis of 12 grazing plots using random sampling method. Different lowercase letters indicate that grand means differ significantly between the grazing treatments (*p* < .05). CK, no grazing; HG, heavy grazing; LG, light grazing; MG, moderate grazing.

Height of *C. songorica* decreased with increased grazing intensity, and there were significant differences between CK and MG, CK and HG, and LG and HG treatment plots (Figure 3A, *F* = 9.81, *p* < .05). The coverage of *C. songorica* decreased with increased grazing intensity, and there were significant differences between CK and HG treatment plots (Figure 3B, *F* = 2.92, *p* < .05). The density of *C. songorica* first increased and then decreased with increased grazing intensity, and there was no significant difference between treatment plots (Figure 3C, *F* = 1.87). The standing crop of *C. songorica* decreased with increased grazing intensity, and there was no significant difference between treatment plots (Figure 3D, *F* = 1.14). The main reason for this was that the 10 quadrats selected in the experimental plot had large degrees of randomness and change and large spatial distribution patches. Therefore, the mean values of *C. songorica* density and standing crop were different, but there was no difference in the variance analysis. In general, with increased grazing intensity, the height, coverage, and standing crop of *C. songorica* decreased in a gradient manner. Although the density of *C. songorica* in HG decreased, it showed an overall upward trend. Heavy grazing (HG plot) resulted in decreased height, coverage and standing crop, and increased density.

### 3.2 Responses of *Cleistogenes songorica* density to grazing intensity and spatial scale

Grazing intensity, spatial scale, and the interaction between them had significant effects on the density of *C. songorica* plant population (Table 1, *p* < .05). The spatial scale factor had a large difference in the square of the density of *C. songorica*, indicating that the change in density of *C. songorica* was more discrete under the spatial scale factor.

**TABLE 1:** The effects of interaction between grazing intensity and spatial scale on *Cleistogenes songorica* density.

Source	df	SS	MS	*F* value	*p* Value
Model	6	4.6989	0.7832	190.20	<.0001
Grazing intensity	3	0.1176	0.0392	9.52	.0037
Scale	3	4.5813	1.5271	370.88	<.0001
Error	9	0.0371	0.0041		
Corrected total	15	4.7360			

**FIGURE 4:**
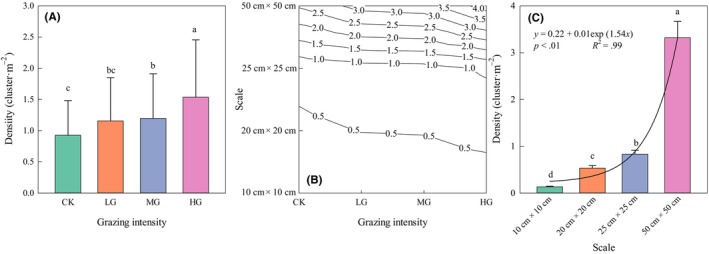
Effects of sampling unit on density of *Cleistogenes songorica*. Mean values ± SE (*n* = 4). The results were the variance analysis of 5 m × 5 m plots by mechanical sampling method. Different lowercase letters indicate that grand means differ significantly between spatial scales (*p* < .05). CK, no grazing; HG, heavy grazing; LG, light grazing; MG, moderate grazing.

The changes in *C. songorica* density under different grazing intensities are shown in Figure 4A. The density of *C. songorica* showed an upward trend under different grazing intensities, and the overall increase was small. There were significant differences between CK and MG, CK and HG, LG and HG, and MG and HG treatment plots (*p* < .05). Unlike the density in Figure 3C, the density in the 5 m × 5 m plot in the analysis of variance is a very large sample, which can better reflect the true state of the density of *C. songorica*. Figure 4B shows the density changes of *C. songorica* under different grazing intensities and spatial scales. This illustrates that the density of *C. songorica* increases with increasing grazing intensity and spatial scale, and change is most obvious between 25 cm × 25 cm and 50 cm × 50 cm. Density of *C. songorica* exponential increased from 25 cm × 25 cm to 50 cm × 50 cm with significant differences at each spatial scale (Figure 4C, *p* < .05). There was an exponential relationship between the spatial scale and density of *C. songorica*, and the goodness of fit was good. The formula was *y* = 0.22 + 0.01exp (1.54*x*).

### 3.3 The semi‐variance function of *Cleistogenes songorica* density under different grazing intensities and spatial scales

In the CK treatment plot, the exponential and spherical models showed the best fitting effect at scales of 10 cm × 10 cm, 20 cm × 20 cm, 25 cm × 25 cm, and 50 cm × 50 cm, and their residual values were small (Table 2a). In the analysis of semi‐variance function, the spatial variation was caused by random factors. That is, the nugget value *C*_0_ is the largest at the scale of 25 cm × 25 cm, while the maximum spatial variation *C*_0_ + *C* = 6.99 and the maximum structural ratio (*C*/(*C*_0_ + *C*) = 99.86%) appear at the spatial scale of 50 cm × 50 cm, indicating it is mainly controlled by structural factors. With increased spatial scale, the values of *C. songorica* spatial auto‐correlation were 6.50, 12.00, 16.00, and 92.50 cm. The spatial auto‐correlation scale *A*_0_ increases with increased spatial scale, indicating that the spatial heterogeneity of *C. songorica* decreases as spatial scale increases.

**TABLE 2:** The curve‐fitted semi‐variograms at different scales under different grazing intensity treatments.

Grazing intensity	Scale	Model fit		Semi‐variance parameters			Auto‐correlation	
		Model	RSS	*C*_0_	*C*_0_ + *C*	*C*/(*C*_0_ + *C*) (%)	Range *A*	*A*_0_
CK(a)	10 cm × 10 cm (1)	Exponential	3.51 × 10^−5^	0.02	0.13	89.55	1.95	0.65
	20 cm × 20 cm (2)	Exponential	2.05 × 10^−3^	0.06	0.66	90.26	1.80	0.60
	25 cm × 25 cm (3)	Exponential	6.78 × 10^−3^	0.14	1.25	89.17	1.92	0.64
	50 cm × 50 cm (4)	Spherical	1.29 × 10^−1^	0.01	6.99	99.86	1.85	1.85
LG(b)	10 cm × 10 cm (1)	Exponential	6.82 × 10^−5^	0.01	0.14	90.57	1.20	0.40
	20 cm × 20 cm (2)	Exponential	4.75 × 10^−3^	0.05	0.63	92.58	1.26	0.42
	25 cm × 25 cm (3)	Exponential	1.72 × 10^−2^	0.05	1.15	95.32	1.41	0.47
	50 cm × 50 cm (4)	Exponential	3.33 × 10^−1^	0.01	5.61	99.82	1.86	0.62
MG(c)	10 cm × 10 cm (1)	Exponential	8.78 × 10^−5^	0.02	0.15	85.81	2.13	0.71
	20 cm × 20 cm (2)	Exponential	2.70 × 10^−3^	0.11	0.72	84.50	2.04	0.68
	25 cm × 25 cm (3)	Exponential	3.94 × 10^−3^	0.18	1.32	86.32	1.86	0.62
	50 cm × 50 cm (4)	Spherical	1.67 × 10^−1^	0.68	8.57	92.06	1.70	1.70
HG(d)	10 cm × 10 cm (1)	Exponential	8.99 × 10^−4^	0.01	0.20	92.89	1.80	0.60
	20 cm × 20 cm (2)	Exponential	1.42 × 10^−1^	0.08	1.02	92.67	2.28	0.76
	25 cm × 25 cm (3)	Exponential	3.86 × 10^−2^	1.25	3.78	66.97	92.97	30.00
	50 cm × 50 cm (4)	Gaussian	9.70 × 10^−2^	7.29	18.55	60.70	7.38	4.26

Abbreviations: CK, no grazing; HG, heavy grazing; LG, light grazing; MG, moderate grazing.

In the LG treatment, the best‐fitting model of the semi‐variance function is exponential, and its residual value is small (Table 2b). In the semi‐variance function analysis, the largest spatial variation of 0.05 was at 25 cm × 25 cm and was caused by random factors. At the spatial scales of 10 cm × 10 cm, 20 cm × 20 cm, 25 cm × 25 cm, and 50 cm × 50 cm, the spatial auto‐correlation of *C. songorica* was 4.00, 8.40, 11.75, and 31.00 cm, respectively. The scale and range of spatial auto‐correlation increase with increased spatial scale, and the maximum spatial variation *C*_0_ + *C*, structural ratio *C*/(*C*_0_ + *C*), and spatial auto‐correlation *A*_0_ were largest at the spatial scale of 50 cm × 50 cm. This indicates that spatial heterogeneity of *C. songorica* decreased with increased spatial scale and that spatial variation was mainly controlled by structural factors.

In the MG treatment, the optimal model of semi‐variance function fitting is exponential and spherical, and the residual value is small (Table 2c). In the semi‐variance function analysis, the spatial variation *C*_0_, spatial maximum variation *C*_0_ + *C*, and structural ratio *C*/(*C*_0_ + *C*) caused by random factors increase with increased spatial scale and are mainly affected by structural factors. Values of spatial auto‐correlation with increasing spatial scale were 7.10, 13.60, 15.50, and 85.00 cm. While the range of spatial auto‐correlation increases with increased spatial scale, its floating value is small. Overall, spatial heterogeneity of *C. songorica* decreased with increased spatial scale.

In the HG treatment, the exponential and Gaussian models have the best fit at various spatial scales, and their residual values are small (Table 2d). In the semi‐variance function analysis, the spatial variation *C*_0_ and the maximum spatial variation *C*_0_ + *C* caused by random factors increase with increased spatial scale, and the structural ratio *C*/(*C*_0_ + *C*) decreases with increased spatial scale until the minimum value is reached at 60.70%. The spatial variation is mainly controlled by structural factors. Spatial auto‐correlation at the scales of 10 cm × 10 cm, 20 cm × 20 cm, 25 cm × 25 cm, and 50 cm × 50 cm was 6.00, 15.20, 750.00, and 213.00 cm, respectively. The 25 cm × 25 cm scale showed the highest spatial auto‐correlation, indicating that the *C. songorica* plaque phenomenon is most apparent at the 25 cm × 25 cm scale. In general, spatial heterogeneity of *C. songorica* decreased with increasing spatial scale (except at 25 cm × 25 cm scale).

### 3.4 Spatial distribution of *Cleistogenes songorica* density at different grazing intensities and spatial scales

**FIGURE 5:**
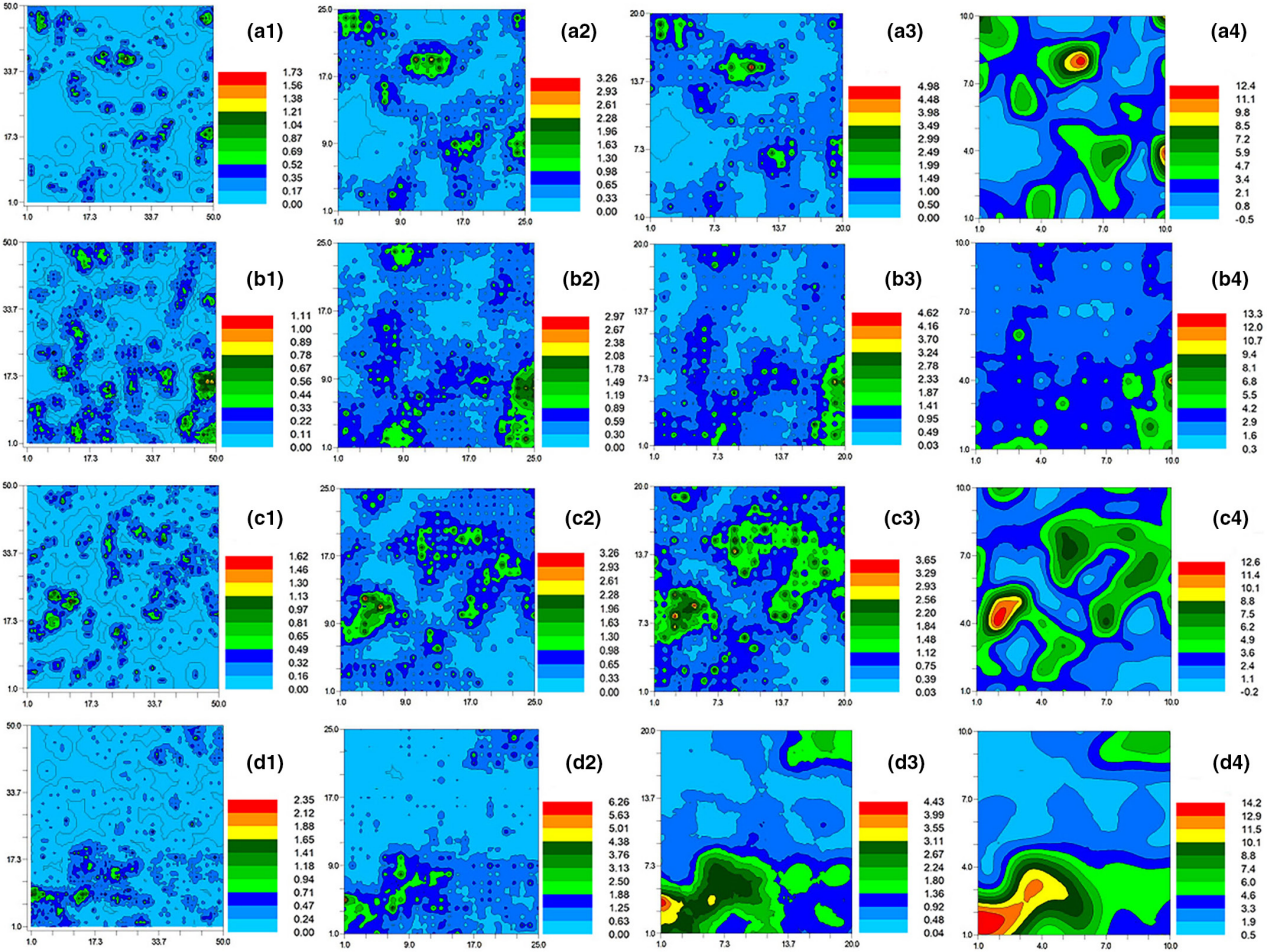
The two‐dimensional spatial pattern of *Cleistogenes songorica* under different grazing intensities. (1) 10 cm × 10 cm; (2) 20 cm × 20 cm; (3) 25 cm × 25 cm; and (4) 50 cm × 50 cm. (a) CK, no grazing; (b) LG, light grazing; (c) MG, moderate grazing; (d) HG, heavy grazing. Different color bands represent the range of different interpolations, and number values represent population density.

The two‐dimensional spatial distribution pattern of *C. songorica* can reflect its stage of degradation succession under stress of grazing intensity (Figure 5). According to the Kriging interpolation method, the spatial distribution map shows the heterogeneity and complexity of the spatial distribution of *C. songorica*, including distribution characteristics of patchiness, gradient and mosaic (Lv et al., 2019). As grazing intensity increased, the spatial distribution of *C. songorica* changed from small‐area to large‐area patch distribution and eventually banded distribution. The aggregation in plots CK and LG was stronger and mainly included *C. songorica* mother plants. The scattered points in MG and HG plots were more intensive, with *C. songorica* divided from a complete plant cluster into several smaller, isolated clusters with obvious fragmentation. Overall, the number of patches gradually decreased with increased grazing intensity and spatial scale while patch area gradually increased and tended toward zonal distribution. With increased grazing intensity and spatial scale, mother plants of *C. songorica* split into several smaller, isolated clusters, a pattern which led to the overall fragmentation of *C. songorica* under heavy grazing.

## 4 DISCUSSION

Grazing is one of the main disturbance factors in the grassland ecosystem, and its impact is complex (Matos et al., 2020). Grazing has both positive and negative effects on the growth and reproduction of plant populations on the grassland. The kinds of compensatory growth mechanisms adopted by plants after being grazed is related to grazing system, grazing intensity, grazing time and physiological and ecological adaptability of plants (Chieppa et al., 2019; Liu, Wang, et al., 2020; Su et al., 2019).

Grazing first affects the quantitative characteristics of grassland plant populations but its degree of influence differs by grazing intensity. The results of this study showed that, under increased grazing intensity, *C. songorica* height, coverage, and standing crop decreased, while density increased. Holechek et al. (2006) found that the standing crop under heavy grazing was lower than that under moderate and light grazing, this is consistent with the results of this study. While increased feeding intensity provides livestock with excellent forage with good palatability, grazing intensity that exceeds the rate most suitable for a grassland will cause changes in individual plant morphology (Moretto & Distel, 1997). Studies have confirmed the phenomenon of overcompensated growth in *C. songorica*, and some degree of animal feeding and trampling can stimulate the growth of new *C. songorica* leaves (Lv et al., 2019; McIntire & Hik, 2002). To adapt to high‐intensity livestock feeding, plant populations will reduce leaf area and growth rate to reduce losses caused by livestock feeding, as well as shrink toward low energy, show strong “grazing avoidance” ability, and sacrifice the competitiveness of the vertical dimension rather than the horizontal dimension (Adler et al., 2005; Stone & Weaver, 2003). For example, height decreases to minimize the standing crop vulnerable to livestock feeding, creating greater opportunity for survival. Individuals are also allocated more resources for producing vegetative organs and seeds to compensate for the damage caused by grazing and ensure the continuation of populations (Liu et al., 2019; Peng et al., 2013; Suzuki & Suzuki, 2011).

Under the disturbance of grazing, *C. songorica* populations will show certain changes in different gradients. In this study, grazing intensity and spatial scale increased the density of *C. songoric* at a relatively flat rate of increase. Increased density in the LG plot was small, indicating that mild interference was beneficial to the abundance of *C. songorica* and richness of the plant population. The density of *C. songorica* increased exponentially under the influence of spatial scale, indicating that the plant population was more sensitive to this parameter. Frequent trampling by livestock will drive division of a complete *C. songorica* cluster into several smaller clusters, thereby increasing the population density (Mooney et al., 2010; Peng et al., 2013).

The spatial distribution of *C. songorica* shows that its spatial heterogeneity will decrease with increased grazing intensity and spatial scale, and will be affected by structural factors. These factors are mainly determined by the spatial heterogeneity of soil resources in desert steppe (Laycock, 1991). The formation of spatially distributed patches and differences in nutrient content determine the degree of variation of *C. songorica* spatial distribution. Because *C. songorica* is a perennial sparse cluster grass, each tillering node of the mother plant produces its own tillering in a growing season. The tillering layer can also be a tillering, and may ultimately form a tillering cluster (Conti & Díaz, 2013; Lv et al., 2019). In this experiment, the spatial scale of 25 cm × 25 cm in the HG plot is unique as it shows a phenomenon of super‐large patches. This may be because in the semiarid grassland ecosystem, the water enriched by vegetation patches that remain after grazing may promote plant growth in those patches, resulting in the irreversible development of large‐scale grassland vegetation composition with more obvious patching (van de Koppel et al., 2002).

The size of spatial scale plays an important role in the spatial pattern and heterogeneity of *C. songorica* (Liu, Zhang, et al., 2018). Habitat fragmentation is one of the main reasons for biodiversity and ecosystem degradation. Habitat fragmentation has long existed in desert steppe, showing obvious plaque characteristics (Lin et al., 2010). In this study, the number of patches gradually decreased with increased grazing intensity and spatial scale while the area of patches gradually increased and tended toward banded distribution and fragmentation of plant clusters.

In desert steppe, previous studies have shown that there are differences in the dimensions and combinations of stability and adaptability of *S. breviflora* under different grazing intensities. With the increase of grazing intensity, the correlation between density and standing crop of *S. breviflora* decreases, and the phenomenon of plant cluster fragmentation occurred (Lv et al., 2020). With the increase of grazing intensity, the spatial heterogeneity of density and height of *A. frigida* decreased (Wang et al., 2022). In this study, *C. songorica* as the dominant species in the desert steppe, with the increase of grazing intensity, the phenomenon of plant cluster fragmentation appeared. The main reasons are: Although it is generally difficult for livestock to crush sparse grasses, this experiment focused on the desert steppe of Inner Mongolia. Here, bare surface area increases due to excessive trampling of livestock. Wind and soil erosion lead to bare tillering nodes and result in the fragmentation of *C. songorica*. The seed dispersal characteristics of *C. songorica* mother plants will form the same aggregation distribution in a limited diffusion range (Chave & Leigh, 2002). This is consistent with the results of Liu, Sun, et al.'s (2020) study on *S. breviflora*. The aggregation distribution of the two plants may be an ecological strategy that offers competitive advantage in homogeneous environments with high spatial aggregation and is thus conducive to the survival of populations under heavy grazing (Saiz et al., 2016). This model enhanced plants' ability to settle, compete, and recover from herbivorous predation in different habitats (Schmid et al., 1988), thus forming a “safe island.”

Grazing affects plant growth, degrades the physical structure of soil, and reduces water permeability of soil, thereby affecting soil spatial distribution (Chen et al., 2013; Liu, Lü, et al., 2018; Yavuz & Karadag, 2015). Studies have shown that plants can preferentially find nutrients in “exotic” soil patches in spatially heterogeneous soils, which may reduce imbalance of inter‐species competition (Hendriks et al., 2015; Xue et al., 2018). However, in soil with spatial heterogeneity of a single plant population, competitive individuals will adopt the same strategy and yield poor performance as a result (Bennett et al., 2017; Burns & Brandt, 2014; Teste et al., 2017). Changing spatial heterogeneity of vegetation with increasing spatial scale is likely to be driven by spatial heterogeneity of terrain characteristics and soil properties in which the vegetation is located, a process which also affects biodiversity of plant communities (Xu et al., 2000; Zuo et al., 2012). Therefore, in order to better understand how spatial heterogeneity responds to spatial scale and grazing intensity, future research should explore the internal mechanism of vegetation spatial heterogeneity.

## 5 CONCLUSIONS

The results of this study showed that both grazing intensity and spatial scale increased the density of *C. songorica* in desert steppe of Inner Mongolia, with spatial scale causing more obvious changes. The spatial distribution of *C. songorica* was affected by structural factors. With increased grazing intensity and spatial scale, the number of patches decreased, and patch areas increased. Heavy grazing resulted in the fragmentation of *C. songorica* plant clusters and promoted the dispersal of *C. songorica* mother plants into smaller clusters, thus increasing grazing tolerance to be more suitable for survival. Grazing intensity and spatial scale have a strong regulating effect on the spatial pattern of *C. songorica* in desert steppe, which may further affect the stability and sustainable development of grassland ecosystems.

## AUTHOR CONTRIBUTIONS

**Zihan Wang:** Data curation (equal); writing – review and editing (equal). **Shijie Lv:** Writing – original draft (equal); writing – review and editing (equal). **Hongmei Liu:** Data curation (equal). **Chen Chen:** Investigation (equal). **Zhiguo Li:** Supervision (equal). **Zhongwu Wang:** Funding acquisition (equal). **Guodong Han:** Data curation (equal); supervision (equal).

## FUNDING INFORMATION

This work was supported by the Interdisciplinary Fund Project of Inner Mongolia Agricultural University (BR22‐14‐04), National Natural Science Foundation of China (31760143, 32260352), Key Project of Science and Technology in Inner Mongolia of China (2021ZD0044), and Inner Mongolia Autonomous Region Natural Science Foundation project (2021MS03042).

## CONFLICT OF INTEREST STATEMENT

The authors declare that they have no known competing financial interests or personal relationships that could have appeared to influence the work reported in this paper.

## Supporting information


Data S1.

Data S2.

## Data Availability

The data that supports the findings of this study are available in the supplementary material of this article. Data sharing is not applicable to this article as no new data were created or analyzed in this study.
