# Supplementary material for: Heavy grazing causes plant cluster fragmentation of sparse grasses
Source: Ecol Evol. 2023 Oct 5;13(10):e10581. doi: 10.1002/ece3.10581 (PMC10551740; doi:10.1002/ece3.10581)
Supplement: Supplementary file 1 — Data S1. [file ECE3-13-e10581-s001.doc]

**Comparison of numerical values under different grazing intensities：**

**DATA** FF;

DO CHF=**1** TO **3**;

DO CHL=**1** TO **4**;

INPUT YY@@;OUTPUT;

END;

END;

CARDS;

13.94

11.14

15.94

13.73

14.37

24.41

22.09

7.21

42.83

23.91

17.18

9.04

;

**PROC** **ANOVA**;

CLASS CHL;

MODEL YY=CHL;

MEANS CHL/DUNCAN ALPHA=**0.05** hovtest;

**RUN**;

**PROC** **MEANS** MEAN STDERR;

CLASS CHL;

VAR YY;

**RUN**;

**Comparison of numerical values at different scales：**

**DATA** HH;

DO CHL=**1** TO **5**;

DO CHD=**1** TO **4**;

INPUT YY@@;OUTPUT;

END;

END;

CARDS;

0.3200 0.6400 0.8000 1.6000

0.3572 0.7144 0.8930 1.7861

0.3633 0.7266 0.9083 1.8166

0.4118 0.8237 1.0296 2.0591

;

**PROC** **ANOVA**;

CLASS CHL CHD;

MODEL YY=CHL CHD;

MEANS CHL CHD/DUNCAN ALPHA=**0.05**;

**RUN**;

**PROC** **MEANS** MEAN STDERR;

CLASS CHL;

VAR YY;

**RUN**;

**PROC** **MEANS** MEAN STDERR;

CLASS CHD;

VAR YY;

**RUN**;
